# Robust Characterization of the Uterine Myoelectrical Activity in Different Obstetric Scenarios

**DOI:** 10.3390/e22070743

**Published:** 2020-07-05

**Authors:** Javier Mas-Cabo, Yiyao Ye-Lin, Javier Garcia-Casado, Alba Díaz-Martinez, Alfredo Perales-Marin, Rogelio Monfort-Ortiz, Alba Roca-Prats, Ángel López-Corral, Gema Prats-Boluda

**Affiliations:** 1 Centro de Investigación e Innovación en Bioingeniería, Universitat Politècnica de València, 46022 Valencia, Spain; jmas@ci2b.upv.es (J.M.-C.); yiye@ci2b.upv.es (Y.Y.-L.); jgarciac@ci2b.upv.es (J.G.-C.); adiaz@ci2b.upv.es (A.D.-M.); 2 Servicio de Obstetricia, H.U. P. La Fe, 46026 Valencia, Spain; Perales_alf@gva.es (A.P.-M.); monfort_isaort@gva.es (R.M.-O.); roca_alb@gva.es (A.R.-P.); lopez_ang@gva.es (Á.L.-C.)

**Keywords:** Electrohysterogram (EHG), myoelectric uterine activity, postpartum, spectral content, sample entropy, spectral entropy, Lempel–Ziv, Time-reversibility

## Abstract

Electrohysterography (EHG) has been shown to provide relevant information on uterine activity and could be used for predicting preterm labor and identifying other maternal fetal risks. The extraction of high-quality robust features is a key factor in achieving satisfactory prediction systems from EHG. Temporal, spectral, and non-linear EHG parameters have been computed to characterize EHG signals, sometimes obtaining controversial results, especially for non-linear parameters. The goal of this work was to assess the performance of EHG parameters in identifying those robust enough for uterine electrophysiological characterization. EHG signals were picked up in different obstetric scenarios: antepartum, including women who delivered on term, labor, and post-partum. The results revealed that the 10th and 90th percentiles, for parameters with falling and rising trends as labor approaches, respectively, differentiate between these obstetric scenarios better than median analysis window values. Root-mean-square amplitude, spectral decile 3, and spectral moment ratio showed consistent tendencies for the different obstetric scenarios as well as non-linear parameters: Lempel–Ziv, sample entropy, spectral entropy, and SD1/SD2 when computed in the fast wave high bandwidth. These findings would make it possible to extract high quality and robust EHG features to improve computer-aided assessment tools for pregnancy, labor, and postpartum progress and identify maternal fetal risks.

## 1. Introduction

There are as yet no reliable tools available for obstetric services to assist clinical staff in making the right decisions regarding the management and planning of the final phase of pregnancy and labor. This is of special relevance in habitual maternal fetal risk scenarios, such as preterm delivery or postpartum hemorrhage. Preterm labor is labor that occurs before 37 weeks of pregnancy, with a prevalence over 10% of births worldwide [1]. It is the principal cause of death in the first weeks of life and produces long-term morbidities and disabilities for survivors, such as mental retardation, cerebral palsy, attention deficit disorder, or asthma [2,3]. Early diagnosis is the key for preterm birth prevention so as to be able to administer agents that inhibit uterine contractile activity, and when necessary, those that stimulate fetal maturation. Markers such as cervical length or fetal fibronectin have shown some utility in predicting preterm birth. However, these markers provide a highly negative predictive value, while their positive predictive values are quite low and do not identify patients who will deliver preterm [4].

Postpartum hemorrhage (PPH) is another important issue in obstetrics. With a prevalence of about 2–6% of total births in developed countries [5], PPH is one of the main causes of maternal morbidity and mortality and kills around 250 thousand women every year, while between 80% and 90% of cases are caused by uterine atony [6,7] which is related to the inability of the uterus to contract after expelling the fetus.

Electrohysterography (EHG) measures externally the electrical activity associated with the contraction of the myometrial cells in the uterus [4,8]. EHG is made up of two different components: the slow wave, which is related to uterine contractions, and the fast wave which is usually subdivided into two components: fast wave low, which has been associated with signal propagation and fast wave high, which is related to cell excitability. Most authors focus on the fast wave, since the slow wave has only been observed in abdominal recordings, which makes its physiological meaning dubious. The fast wave low of human EHG ranges from 0.13 to 0.26 Hz, and fast wave high ranges from 0.36 to 0.88 Hz, although its energy can be extended up to 4 Hz [9,10]. Indeed, it is common in the literature to analyze the fast wave high component from 0.34 Hz and many of these studies ignore the FWL and focus on the 0.34 to 1 Hz bandwidth. This is because as labor approaches EHG-burst spectral content shifts to the higher frequencies [11] in which breathing and cardiac interference are minimized [12]. 

Previous works revealed that EHG not only surpasses traditional tocography for monitoring uterine dynamics during pregnancy, especially in obese women [8,13,14,15], but also provides additional information on the uterine condition. Its electrical activity is infrequent and uncoordinated in early gestation and as labor approaches it becomes more intense and synchronized [4,16], so that EHG characteristics change throughout pregnancy [16,17]. We recently showed the possibility of monitoring postpartum uterine myoelectric activity, which can be used for the early detection of uterine atony [18].

Several studies have focused on using EHG parameters to identify effective labor contractions in term and preterm pregnancies [10,12,19], even in women with threatened preterm labor undergoing tocolytic therapies [20,21]. Temporal parameters which measure the intensity of contractions such as peak-to-peak amplitude or RMS, and spectral parameters to determine the shift of spectral content to high frequencies when labor approaches, such as mean, median, or dominant frequency, have been used to characterize EHG signals [22]. Lately, the complexity and regularity of EHG signals have been assessed by means of non-linear parameters such as sample entropy, spectral entropy [21], time reversibility [23], or the Lempel–Ziv index [24].

The literature has reported controversial results for EHG parameters and contradictory trends have been identified for temporal, spectral, complexity and regularity, when comparing different obstetric scenarios [25]. Some authors found that EHG amplitude was higher in recordings from women who delivered preterm compared to term [20,26,27], while others found no differences between these groups [19,28]. The frequency-related parameters were worked out as being expected to be more robust to the electrode position and interpatient variability than those related to amplitude [22]. Lucovnik et al. stated that dominant (or peak) frequency was higher for women in preterm labor than preterm nonlabor and were useful for differentiating patients who delivered within seven days from those delivered after seven days [29]. Other studies suggest that the median frequency could be used to discriminate term from preterm labor [19,30]. However, the shift towards higher frequencies seems to occur four days before labor in preterm patients and about 24 h before delivery in term cases [31]. If we broaden the labor prediction time horizon, the trends are not so clear [31,32]. Some works found that sample entropy decreases when labor is near [19], while others reported higher values in the transition from latent to active phase of labor [33].

No significant differences in this parameter have been obtained between term and preterm records [28] or in discriminating imminent labor (<7 days) [21]. for a study of the Lempel–Ziv index showed significantly higher values of this parameter in the range of 0.24–4 Hz for labor in less than seven days [24], while others found that it was in the 0.1–4 Hz range and decreases as labor approaches, pointing out that the EHG becomes more deterministic [21].Time reversibility was able to differentiate labor from non-labor contractions by the regularity parameters, with higher values in labor [23]. This trend was also observed in EHG recordings obtained in women with threatened preterm labor, with significant differences between labor in less vs. more than seven days. However another study revealed that time reversibility was unable to identify imminent labor or preterm deliveries [34].

These discrepancies could be attributable to various factors. Firstly, there is no recognized standard for EHG recordings, different recording protocols with different numbers of electrodes, positions or inter-electrode distances are used. Also, there are differences in the bandwidth (whole EHG bandwidth or fast wave high component) and the signal segments (EHG burst associated with uterine contractions or whole EHG window containing basal and contractile activity) used to compute EHG.

The aim of the present work was to determine the high-quality and robust temporal, spectral and non-linear EHG parameters so as to improve the performance of computer-aided prediction systems for identifying maternal-fetal risks. For this, we assessed the consistency of tendencies according to the proximity of labor in different obstetric scenarios. Using our own (Ci2B-La Fe) database, we analyzed different EHG parameters from pregnancy recordings in women who delivered at term, intrapartum recordings during active phase of labor, and postpartum EHG recordings. We compared the trends of these parameters in between these obstetric scenarios with respect to those obtained in EHG signals from term and preterm deliveries recorded in women during regular pregnancy checkups (public Term-Preterm EHG Database available in Physionet).

## 2. Materials and Methods 

### 2.1. Term-Preterm EHG Database

The “Term-Preterm EHG Database” (TPEHG) is a publicly available database which includes 30-min EHG recordings taken during regular checkups during the third trimester of pregnancy (gestational ages between 22 and 36 weeks) [19]. It contains 300 EHG recordings from 262 women who delivered at term (term group), with a time-to-delivery (TTD) of 92.1 ± 29.4 days and 38 women who delivered prematurely (preterm group) with a time-to-delivery of 53.9 ± 30.1 days. Each session contains multichannel EHG recordings of three bipolar signals (S1, S2 and S3). As Channel S3 usually outperforms other channels in distinguishing term and preterm deliveries [19] we only used this channel in the present study. Channel S3 was obtained as the difference between two electrodes placed in a horizontal row under the navel and keeping an interelectrode distance of 7 cm.

S3 bipolar signals were filtered in the range 0.1 to 4 Hz by using a zero-phase shift 5th order Butterworth bandpass filter, since this bandwidth comprises the main content of EHG signals [9]. Motion-artifact segments were visually identified by three different experts and discarded from the study. In case of disagreement, the decision was taken through a blind majority vote based on their reports. In this regard, the criteria adopted to discard EHG sections were significant and abruptly increased the amplitude of non-physiological events with respect to the basal tone, and also the identification of respiratory interference associated with the appearance of “sinusoidal” components with a frequency between 12 and 24cpm (0.2–0.4 Hz).

### 2.2. Ci2B-La Fe Database

**Figure 1:**
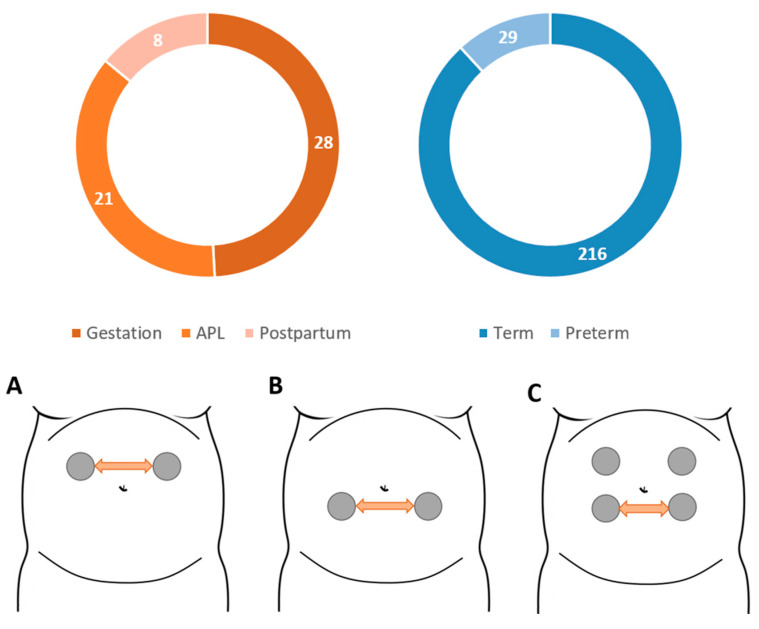
EHG recordings distribution according to the obstetric scenario in which they were obtained for the Ci2B-La Fe and TPEHG databases (Upper figure) and the different recording protocols associated with each obstetric scenario (Lower figure). Protocol A is for the Active phase of labor and Gestation recordings. Trace B is used for the Postpartum group and trace C for the TPEHGDB. Note that 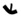 represents the patient’s navel.

This database was composed of 57 EHG recordings with durations between 30 min to 1h, taken in three obstetric scenarios in the Hospital Universitari i Politècnic La Fe in Valencia, whose Institutional Review Board approved the procedure. The first (Gestation) group contains 28 recordings from pregnant women (gestational ages between 25 and 36 weeks) threatened with preterm labor who finally delivered at term, with a TTD of 37.5 ± 28.7 days. The second (APL) group contains 21 recordings from term labor women during active labor. The differences between the gestation and APL groups made it possible to study the trend of different EHG parameters as labor approaches. The third (postpartum) group contains 8 recordings from the first 3 h after labor in women who initiated labor spontaneously and delivered vaginally at term. All the patients were informed of the study and gave their prior written informed consent. Signals were acquired by placing two disposable Ag/AgCl electrodes (3M red dot 2560) electrodes on the maternal abdominal surface symmetrically positioned with respect to the median axis over the umbilicus keeping an interelectrode distance of 8 cm. Two additional electrodes on the hips were used for signal reference and ground. Due to the fact that the uterine volume and position changes through pregnancy, the optimal electrode position for recording uterine muscle may change between obstetric situations, as in the case of Gestation & APL vs. postpartum groups [35]. Figure 1 summarizes the distribution of the EHG recordings used according to the obstetric scenario in the upper trace, and the different recording protocols associated with each group. In all the databases used, only one recording was carried out on each patient, except in two patients in the gestation group in the Ci2B-La Fe database, in which two recordings were taken.

The two monopolar signals recorded in each scenario were bandpass filtered in the range 0.1–4 Hz using a zero-phase shift 5th order Butterworth filter and sampled at 20 Hz. We subtracted these to obtain a bipolar signal of higher quality. Segments with motion artifacts and severe cases of respiratory interference were identified by experts and excluded from the study as described in Section 2.1. Further details of the recording system and data preprocessing can be found in a previous study [21].

### 2.3. EHG Signal Characterization

We performed a whole window analysis to extract different features from the EHG signals, using 120 s analysis windows with a 50% overlap to keep EHG sections representative at a reasonable computational cost [21]. This type of analysis does not require annotating each signal burst, which can be tedious and subjective, and is more suitable for future ‘real-time’ applications. For each window analysis, a set of temporal, spectral, and non-linear parameters were computed to characterize EHG signals. The root mean square (RMS) value of the signals was computed in the whole bandwidth (WBW: 0.1–4 Hz) since it is closely related to the intensity of uterine myoelectrical activity [4]. For the spectral parameters, which are related to cell excitability [4,9], we computed the dominant frequency (DF) in the range of 0.34–1 Hz in order to reduce respiration and cardiac interference [36,37]. The deciles of the power spectrum in the WBW were also computed. We only included the results of decile 5 (which is the commonly used median frequency) and deciles 7 and 3, which showed similar results to 6 and 4 respectively, but no remarkable findings were found on the others. We also computed the spectral moment ratio (SMR) which has been proposed by Dimitrov et al. to evaluate muscle fatigue in electromyographic signals by assessing the spectral content shifting (see Equation (1)) [38], which presents lower values as muscular fatigue increases because of the shifting of the spectral content toward lower frequencies.

(1) $$SMR=\ln\frac{M_{-1}}{M_{5}}\;\;$$

where *M*_−1_ and *M*_5_ are the spectral moment of order −1 and 5 respectively (see Equation (2)) [38]:

(2) $$M_{k}=\overset{f_{\max}}{\underset{f_{\min}}{\int }}f^{k}*PS\left(f\right)*df\;\;$$

where *f_min_* and *f_max_* are the signal bandwidth 0.1–4 Hz, *f* represents the vector of frequencies and *PS(f)* is the power spectrum associated with each frequency.

As biological processes are known to involve non-linear behavior, a set of non-linear parameters were selected to estimate signal complexity [39]. In this work we computed the following non-linear parameters which have been widely used for characterizing EHG signals: Lempel-Ziv complexity in its binary form using the median value as threshold [40], sample entropy [41], time reversibility [42], and spectral entropy [43]. We also obtained the Poincare plot of consecutive EHG signal samples since the actual data may be intimately related to past information. We calculated the SD1/SD2 ratio to assess signal randomness, where SD1 and SD2 are the dispersion along the minor and major axes of the ellipse associated with short and long-term EHG signal variation, respectively [44]. Due to the controversial results in the literature, the non-linear features were computed in the WBW and in a more restrictive range which corresponds to the fast wave high (FWH: 0.34–4Hz) components in order to find robust parameters [9].

We then obtained representative values for each parameter in each recording. Taking account of the uterine electrophysiology throughout the pregnancy, the time- percentage of EHG-bursts (associated with uterine contraction) in the recording is expected to be relatively low, especially in pregnancy recordings (maximum contraction rhythm: 3 in 10-min), i.e., the median value of all analyzed windows mainly assesses the basal activity rather than uterine contractions. Since the different EHG parameters may present rising or falling trends as labor approaches, we used the 10th, 50th and 90th percentiles of all the analyzed windows as potential representative values of each recording. The number of analysis windows in each 30 min recording was typically 20–27. The parameter percentiles were computed from these windows per recording session (10th, 50th, and 90th percentiles for each parameter and recording), after which they were clustered for statistical analysis into the groups indicated in Figure 1. We finally compared the trends of each EHG feature in the above mentioned scenarios and determined whether the EHG features presented statistically significant differences between the gestation and APL groups, between APL and postpartum and between term and preterm delivery groups, using the Wilcoxon rank sum test (α = 0.05).

## 3. Results

**Figure 2:**
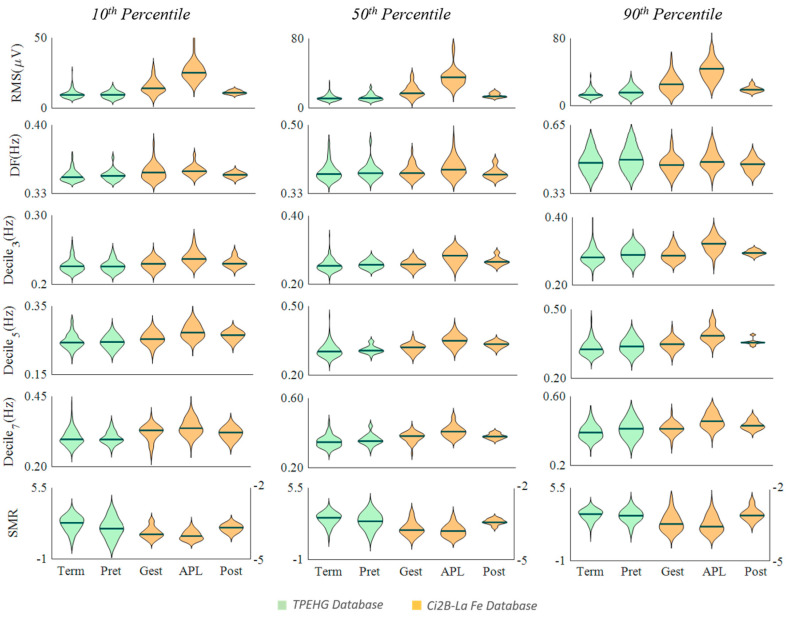
Distribution of temporal and spectral EHG parameters in different obstetric scenarios (Term, Preterm, Gestation, Active phase of labor and Postpartum) for the TPEHG public database (green) and Ci2B-La Fe database (orange). Note that SMR is the abbreviation of spectral moment ratio.

**Figure 3:**
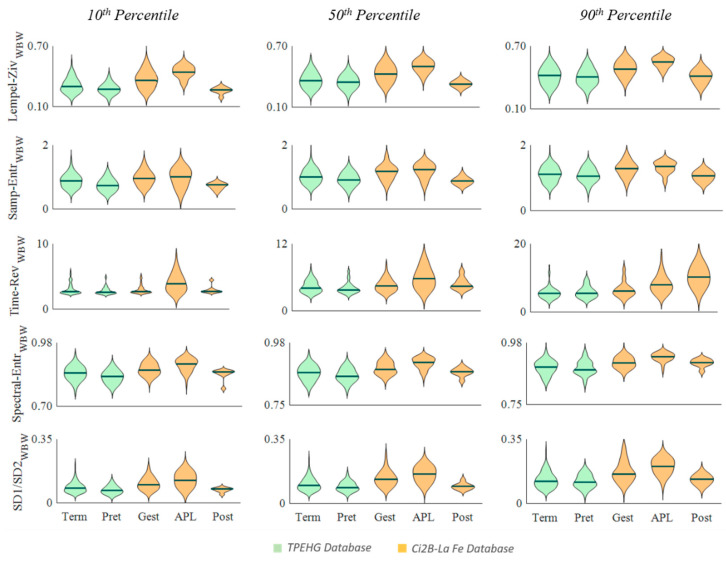
Distribution of non-linear EHG parameters computed in the WBW in the different obstetric scenarios (Term, Preterm, Gestation, Active phase of labor and Postpartum) for the TPEHG public database (green) and Ci2B-La Fe database (orange).

**Figure 4:**
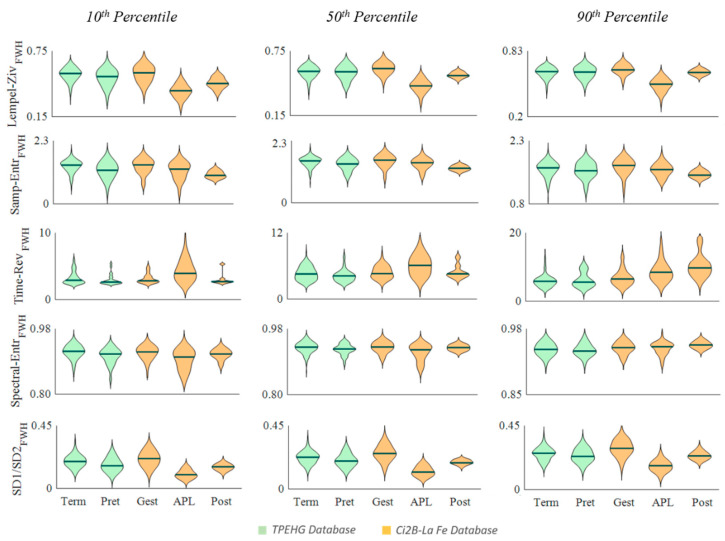
Distribution of non-linear EHG parameters computed in the FWH bandwidth in the different obstetric scenarios (Term, Preterm, Gestation, Active phase of labor and Postpartum) for the TPEHG public database (green) and Ci2B-La Fe database (orange).

Figure 2, Figure 3 and Figure 4 show the distribution of the computed parameters for the different groups from the 10th, 50th, and 90th percentiles to obtain a representative value of each recording. Note that the spectral moment ratio shown in Figure 1 has two vertical scales, one on the left and one on the right for the TPEHG database and the Ci2B-La Fe database, respectively.

Table 1, Table 2 and Table 3 contain the parameter p-values from statistical comparisons of the groups: term vs. preterm, gestation vs. APL, APL vs. postpartum.

Figure 2 shows the results of the temporal and spectral EHG parameters. The Ci2B-La Fe RMS values were maximum during labor and significantly smaller in pregnancy and after delivery at all percentile values used to obtain the representative data (Table 1). Dominant frequency also showed maximum median values during labor, but differences were not significant except when using the 90th percentile in gestation vs. APL. Deciles from the power spectrum showed the same trend as the RMS, and most obtained significant differences when comparing the APL group against the gestation and postpartum groups regardless of the percentile used to obtain the representative value. The TPEHGDB preterm deliveries (closer to labor) obtained slightly higher values for these parameters than term deliveries (farther to labor), but not so remarkable (significant only for RMS and Decile 3 using the 90th percentile), given that both term and preterm recordings were carried out far from labor.

SMR was smaller during the active phase of labor than in gestation (significant differences for 10th percentile) and maximum in postpartum (significant differences in all cases). SMR was also smaller in the group closer to labor (preterm deliveries) in TPEHGDB for all percentiles, obtaining significant differences with term deliveries in the 10th percentile.

For the non-linear parameters computed in the whole bandwidth (Figure 3), Lempel–Ziv, sample entropy, spectral entropy and ratio SD1/SD2, showed a similar trend to RMS and spectral parameters, i.e., increasing from pregnancy to labor and decreasing in the postpartum group for the three percentiles in the Ci2B-La Fe database. Regardless of the percentile used to obtain the representative data of each recording, most of these parameters showed significant differences between the gestation and APL groups, and between APL and postpartum (Table 2). In contrast, these parameters showed a smaller value in the group closer to delivery (preterm) than in the TPEHGDB term group, significant when using the 10th percentile

When Lempel–Ziv, sample entropy, spectral entropy, and ratio SD1/SD2 were computed in the fast wave high bandwidth (Figure 4), they showed a consistent falling trend, as labor was closer in both databases and then increased after delivery. Differences were statistically significant again for most cases in the Ci2B-La Fe, and with the 10th percentile of the TPEHGDB, with some additional cases with the 50th percentile (Table 3). 

Time reversibility showed a significant increase from gestation to labor regardless of the computation bandwidth and the percentile used to obtain the representative value. It also increased after delivery for percentile 90 but dropped in percentiles 50 and 10, differences significant only for this latter. The TPEHGDB barely shows any significant change for this parameter, only for percentile 50 when computing in the FWH bandwidth.

## 4. Discussion

Preterm labor is still one of the major causes of mortality and morbidity in obstetrics. EHG has been emerging as a promising tool to predict preterm labor thanks to its high sensitivity [4]. The extraction of useful information embedded in EHG recordings has become a critical issue in designing computer-aided systems based on the machine learning algorithm for predicting preterm labor, since the quality of features that distinguish real from false preterm labor is vital to achieve satisfactory classifier accuracy [45].

### 4.1. Robust Characterization of Uterine Activity in Whole EHG Recordings Analysis

Firstly, EHG recordings are often contaminated by mother and/or fetal motion artifacts and respiration interference [46], which must be discarded from the study. Although some studies attempted to analyze the whole EHG recording including these non-physiological segments [19,47,48,49], we believe that this may distort the real feature space of the obstetrical scenario analyzed. After eliminating the non-physiological segments from signal analysis, we should extract information from various physiological segments of variable duration. Since the window length may significantly affect the computation of non-linear parameters [50], we preferred to compute the EHG parameter on a moving 2-m window, which is a trade-off between computational cost and information loss [21]. 

In this work we attempted to compare different percentile values (percentile 10, 50, and 90) of all analyzable windows in the recording to obtain robust representative data for each EHG parameter. We also tested other percentile values and we found similar trends for the parameters between percentiles 25 and 10 and between 75 and 90 (results not included for reasons of space).

As shown in results section, we did not obtain a considerable difference for the Ci2B-La Fe database in different percentile values in terms of discriminatory capacity between the gestation and APL groups or between APL and postpartum. This may be related to the fact that EHG signal changes are more evident during the active phase of labor when compared with pregnant women far from delivery. In contrast, when attempting to distinguish subtle changes between term and preterm deliveries in the TPEHG database, the largest discriminatory ability was obtained for percentiles 10 or 90 according to the EHG parameter. Our results showed that the amplitude-related parameter, which has been proven to present a rising trend due to the major recruitment of uterine cells involved in one contraction as labor approaches [4,26], can better discriminate term and preterm deliveries for percentile 90. Similarly, since signal spectral contents shift to higher frequencies as labor progresses [22,37], percentile 90 of decile 3 spectral parameters was shown to better differentiate term and preterm deliveries, while non-linear parameters with a falling trend as delivery approaches [19,21] performed better when using percentile 10 of all analyzable windows as its representative data. Another issue that could help to explain these results is the incidence of uterine contractions. During pregnancy the maximum contraction frequency is three 40–60 s contractions every 10 min [51], which yields a maximum percentage time of the presence of EHG-bursts of about 30%. Considering that uterine contractions present higher amplitude and frequency and lower complexity than basal activity, the better discrimination ability of the 90th percentile for the former and the 10th percentile for the latter also points to a direct relationship between discrimination ability and the fact that the representative value of a parameter is associated with contractile segments.

### 4.2. Temporal & Spectral EHG Parameters

We found that the signal amplitude of our database is higher than that of the TPEHG. This may be due to the possible differences in recording factors such as inter-electrode distance, electrode position, skin preparation, and gestational age at recording. Nevertheless, its rising trend throughout pregnancy, which reflects the underlying increase of uterine cells involved in one contraction [4,51], was observed in both databases (APL > gestation, preterm > term). This RMS difference in term and preterm deliveries was significant for percentile 90, while it was not found to be so in previous studies on the same database when computed on the whole 30-min recording [19] or only on EHG-bursts [28]. This reveals the importance of the computation method in obtaining high quality EHG features.

Dominant frequency is one of the most commonly used spectral parameters to characterize EHG signals and detect labor proximity [22]. Garfield et al. analyzed the DF of EHG-bursts recorded during labor and during gestation, reporting a significant increase in the labor group [36]. Other authors computed DF in different ranges (0.08–4 Hz, 0.3–3 Hz, and 0.3–4 Hz) and only obtained significant differences for the 0.3–4 Hz range when trying to separate term and preterm cases in the TPEHGDB [19]. In this work, even though the DF tended to rise in the groups closer to labor (APL for the Ci2B-La Fe and preterm for the TPEHGDB), we only obtained significant differences when comparing APL and gestation recordings for the 90th percentile, which is in agreement with the results obtained by Garfield et al. [36]. However, DF was not able to differentiate term and preterm groups as reported by Fele-Zorz [19]. These differences may be due to the different bandwidth, which seems to greatly affect this parameter, the computation method, and the exclusion/inclusion of artifacted sections. Considering the results obtained here and those reported by other authors, it seems that DF could be suitable to detect imminent labor but not to detect changes far from labor. This agrees with another study by Garfield et al. which suggests that the shift toward high frequencies would occur about 24 h before labor [52].

Spectral deciles also increased in both databases as labor approaches (APL > gestation, preterm > term), statistically significant differences. This finding is consistent with Fele-Zorz, who found that the median frequency of the fast wave high (computed in the 0.3–3 Hz range) could be used for differentiating preterm and term deliveries [19]. After delivery, uterine cell excitability fell [53], which is reflected in the decrease of spectral deciles, and to a lesser extent, of DF(APL > postpartum).

The spectral moment ratio was proposed years ago to quantify muscle fatigue in the EMG signal [38] and as far as we know this is the first time that it has been used for characterizing EHG. We found that it decreased as labor approaches (APL < antepartum, preterm < term) and increased significantly after delivery. This drop in SMR closer to/during labor suggests that the spectral contents shifted toward higher frequencies due to increased excitation-contraction coupling [38], so that the spectral moment of order 5 increased more than that of order –1, thereby obtaining a lower value for this parameter. The significant increase of this parameter in postpartum is noteworthy, which goes along with the expected uterine muscle fatigue after the contractile efforts during labor.

### 4.3. Non-Linear EHG Parameters

We found controversial results in Ci2B-LaFe database, for whole and FWH bandwidth non-linear parameters. Lempel–Ziv, sample entropy, spectral entropy, and SD1/SD2 computed in FWH bandwidth dropped significantly from pregnancy to labor, suggesting less signal complexity, regularity, and randomness and increased in postpartum. This is consistent with our results from the TPEHG database (less preterm than term complexity), which also agree with Fele-zorz who reported that sample entropy in FWH bandwidth was significantly smaller for the preterm labor group [19]. Vrhovec et al. analyzed sample entropy of EHG-bursts in 0.3–3 Hz in the last two months of gestation (from 32 to 39 weeks of gestation) and found that this parameter significantly drops as labor approaches [33]. They also found that the beginning of labor is characterized by relatively high sample entropy values, which start to fall at the transition from latent phase to APL and achieve relatively low values at childbirth [33]. Our results are also consistent with our previous study in which we found that Lempel–Ziv was significantly lower in women with threatened preterm labor who delivered <7 days than >7 days [21]. Also, for the first time, we reported on the utility of signal randomness measure SD1/SD2 [44] derived from the Poincare plot for discriminating term and preterm deliveries. 

When these parameters were computed in the whole bandwidth their value still decreased close to labor in the TPEHG database but increased from gestation to labor. This latter is consistent with Lemancewicz, who obtained a significantly higher Lempel–Ziv and approximate entropy computed at 0.24–4 Hz for women with threatened preterm labor who delivered in less than seven days than deliveries in more than seven days [24]. In this regard, we decided to compute only the sample entropy since it has been reported to be more independent of recording length and computationally efficient than the approximate entropy [41]. We also believe that the discrepancies with Lemancewicz come from the difference in fast wave low (FWL) components. We hypothesize that no drastic changes of the FWL amplitude during contractions with respect to basal activity occurs in women who are still far from childbirth (term and preterm deliveries in TPEHG and gestation patients in our database), which may lead to a high signal predictability when computing in the whole bandwidth. Nevertheless, since far from labor only sporadic uterine contractions occur, signal complexity computed in the FWH bandwidth is relatively high, which is equivalent to low signal predictability. However, during labor the FWL amplitude would significantly increase, being higher than basal activity, which may increase the signal complexity and reduce its predictability in the whole bandwidth. In FWH, frequent uterine contractions may give rise to lower signal complexity and higher signal predictability than antepartum. Our results suggest that the signal bandwidth in which we computed the non-linear parameters may be a key factor in obtaining a robust and physically interpretable indicator for characterizing EHG signals. We found a consistent trend among different non-linear parameters (Lempel–Ziv, sample entropy, spectral entropy and SD1/SD2) computed in the FWH bandwidth in different obstetric scenarios.

Time reversibility showed an increasing trend as labor approaches in the Ci2B-La Fe database, suggesting higher signal non-linearity. This finding is consistent with other authors, who also stated that time reversibility outperformed sample entropy and spectral parameters for predicting labor [54]. In contrast, we found that time reversibility showed a reverse trend (preterm<term) without a significant difference between term and preterm deliveries in the TPEHGDB, which agrees with other authors who found that this parameter was not reliable for discriminating term and preterm deliveries [34]. We therefore believe that time reversibility may not be sensitive to subtle electrophysiological changes far from delivery and could be more suitable for detecting imminent labor.

Finally, we should point out some limitations of the study. A larger database would be more representative of the different obstetric scenarios analyzed in the present work. We are still working on increasing the number of EHG recordings in different situations, while different parameters may present mutual and/or redundant information. Further work should be carried out to quantify any mutual or redundant information among the parameters in order to obtain high quality EHG features with complementary information to improve computer-aided preterm labor prediction performance. Despite the small size of the databases we believe that our findings are representative of the general population, since they match the physiological changes throughout pregnancy reported in the literature. Indeed, we consider that the present work may help to find optimal parameters and bandwidths that maximize the separability of the different classes of different obstetric scenarios and therefore improve expert system performance.

## 5. Conclusions

In this work, we attempted to analyze the robustness of different parameters for characterizing EHG uterine activity in EHG recordings obtained from different protocols in a variety of obstetric scenarios. Firstly, it should be mentioned that there are very few studies in the literature on postpartum EHG recordings, this being the first time that these signals have been comprehensively studied and contextualized. Secondly, we found that percentiles 90 and 10 in all analyzable windows of the EHG parameters, which are expected to show increasing and decreasing trends throughout pregnancy, respectively, surpass median values in discriminating between obstetric scenarios. Regardless of the database, we found both amplitude-related and spectral parameters (deciles and dominant frequency) increased as labor approaches and then decreased after delivery. In contrast, the spectral moment ratio fell in both databases as labor progresses and then rose afterwards. No consistent trend was found for time reversibility throughout pregnancy, suggesting that this latter is not a reliable indicator for characterizing EHG signals. We also observed a contradictory trend in the non-linear parameters when computed in whole and FWH bandwidth, suggesting that the signal bandwidth may be a key factor in obtaining a robust and physically interpretable indicator for characterizing these signals. Indeed, we found a consistently falling trend as labor approaches among different non-linear parameters (Lempel–Ziv, sample entropy, spectral entropy, and the ratio SD1/SD2) computed in FWH bandwidth in different obstetric scenarios. These findings would make it possible to extract high quality and robust EHG features to improve the discriminatory ability of EHG-based computer-aided systems in different obstetric situations.

## Figures and Tables

**Table 1:** *p*-Values for the temporal and spectral EHG parameters when comparing different obstetric scenarios. Significant differences (*p* < 0.05) are shaded.

	Term vs. Preterm			Gest. vs. APL			Post. vs. APL		
	10th	50th	90th	10th	50th	90th	10th	50th	90th
RMS	0.74	0.61	0.04	<0.01	<0.01	<0.01	<0.01	<0.01	<0.01
DF	0.99	0.68	0.46	0.47	0.08	0.02	0.56	0.14	0.14
Dec3	0.97	0.11	0.04	<0.01	<0.01	<0.01	0.04	0.04	<0.01
Dec5	0.86	0.16	0.07	<0.01	<0.01	<0.01	0.19	0.04	<0.01
Dec7	0.88	0.07	0.06	0.01	<0.01	<0.01	0.06	0.01	0.10
SMR	<0.01	0.13	0.36	0.02	0.13	0.17	<0.01	<0.01	<0.01

**Table 2:** *p*-Values for the non-linear EHG parameters computed in the WBW when comparing different obstetric scenarios. Significant differences (*p* < 0.05) are shaded.

	Term vs. Preterm			Gest. vs. APL			Post. vs. APL		
	10th	50th	90th	10th	50th	90th	10th	50th	90th
Lempel-Ziv	0.03	0.38	0.46	<0.01	<0.01	<0.01	<0.01	<0.01	<0.01
Samp. Entropy	<0.01	0.07	0.49	0.43	0.17	0.21	0.01	<0.01	<0.01
Time Rev	0.09	0.11	0.98	<0.01	<0.01	<0.01	0.04	0.22	0.11
Spec. Entropy	<0.01	0.12	0.57	<0.01	<0.01	<0.01	<0.01	<0.01	<0.01
RatioSD1/SD2	<0.01	0.12	0.56	<0.01	<0.01	<0.01	<0.01	<0.01	<0.01

**Table 3:** *p*-values for the non-linear EHG parameters computed in the FWH bandwidth when comparing different obstetric scenarios. Significant differences (p < 0.05) are shaded.

	Term vs. Preterm			Gest. vs. APL			Post. vs. APL		
	10th	50th	90th	10th	50th	90th	10th	50th	90th
Lempel-Ziv	0.07	0.55	0.91	<0.01	<0.01	<0.01	<0.01	<0.01	<0.01
Samp. Entropy	<0.01	0.05	0.37	0.01	0.01	0.01	0.12	0.010	0.04
Time Rev	0.09	0.04	0.76	<0.01	<0.01	<0.01	0.01	0.08	0.07
Spec. Entropy	<0.01	<0.01	0.82	<0.01	<0.01	0.81	0.18	0.16	0.16
RatioSD1/SD2	<0.01	0.03	0.27	<0.01	<0.01	<0.01	<0.01	<0.01	<0.01
